# The Conditional Grant

**Published:** 1921-12

**Authors:** 


### THE CONDITIONAL GRANT.

**(From a Hospital Secretary.)**

ON January 25 last the Minister of Health, Dr. Addison, appointed a representative Committee to consider the present financial position of the voluntary hospitals and to make recommendations as to any action which should be taken to assist them. The final report, signed by all the members of the Committee, was issued on May 31, 1921. The report, a very comprehensive one, dealt with the whole of the hospitals of the Metropolis, of England and Wales, and of Scotland, and after reviewing the financial condition at the end of 1920, and the effect of increased cost of all requirements in hospitals, and of the increased expenditure on salaries and wages, a unanimous recommendation was made to the Government that £1,000,000 should be allocated to relieve hospitals of their serious temporary difficulties. The general cry for economy was being justly raised throughout the country. It was therefore decided by the Government, as the result of the recommendation of the Lords Committee, to make a grant of £500,000 to meet the absolutely immediate need of the voluntary hospitals of Great Britain, and the administration of this grant was entrusted to a Hospitals Commission. Lord Cave's Committee said: "The position of a large number of hospitals is such as to make it improbable that they can be continued on a voluntary basis unless prompt and vigorous measures are taken to re-establish the position of 1914." Six months have passed, and the majority of hospitals of London (a few have received a small grant) still find themselves crippled by the burdens which had accumulated during the war time, and not one penny of contribution has been received to relieve them of the heavy burden of debt which is crippling their energies.

The Committee did not miss the fact that there is a crying need in many parts of the country for further hospital accommodation, and this is especially the case in the poorer parts of the great cities, in South Wales, and in Scotland. For over seven years there had been little or no provision made to meet the expansion of the population, and it is, the Committee says, plainly impossible under present conditions to raise by public subscriptions the whole of the sums required for this purpose. They recommended that the Hospitals Commission be authorised during the period of two years to recommend grants by Government for the extension or the improvement of hospital accommodation, provided that an equal amount is forthcoming from other sources. It was estimated that a sum of £250,000 may be required for this purpose during the financial year 1921-22—in other words, that a sum of at least £500,000, in addition to the grant of a million pounds already recommended, would be usefully expended during the year 1921-22. As a drowning man grasps at a straw, so the Government appears to have seized this recommendation, and in their decision to give £500,000 in lieu of £1,000,000 added the proviso that grants should only be made on condition that an equal amount of new revenue should be raised. The Government fully accept the view of the Cave Committee that the voluntary system must be maintained. This everyone connected with voluntary hospitals is prepared to work for, but their task is proving one of extraordinary difficulty. Five-and-twenty years ago most of the leading hospitals could number amongst their supporters rich and generous donors, but these, alas! are now so crippled by the present heavy taxation that, willing as they no doubt are to continue to assist their poorer brethren, they cannot do what they have done in the past. Large corporations have absorbed the work of private firms, and these do not recognise their obligations in the generous manner which was traditional amongst the old firms. They must consider—and this they do timidly—what would our shareholders say? In proportion to their means no contributors are more generous than the working men themselves, in spite of the fact that they have their compulsory sickness insurance to meet every week. Some of the hospitals have made a definite charge per week, and are receiving this fixed payment from varying percentages of their patients. Other hospitals make no definite charge, but, putting the exact position of the hospital before all who come and enjoy the benefits of the treatment there, ask for a contribution in proportion to the means of the person.

Lord Cave's Committee calls attention to various schemes which have been tried. In many hospitals in the large manufacturing districts of the North it is not a serious difficulty to raise considerable sums from the working men through their firms. In some of the agricultural districts a system has been adopted of organising regular contributions from the inhabitants and forwarding the sum to the central authority, and the plan adopted by the Radcliffe Infirmary at Oxford has proved a very notable success. Attention was called to what is known as the Sussex Scheme—the National Provident Scheme for Hospital and Additional Medical Service—and a very strenuous attempt is being made, with the approval of King Edward's Hospital Fund, to adapt it to London. At present it appears that those who are sick and need hospital help are applying in considerable numbers for enrolment, whereas large bodies of people in a normal condition of health are still standing aloof. There is a large, and very often a shifting, population of people who are daily workers in the busy life of London, small shops, hotels, offices, etc., and much time must be required to create any effective organisation of any system which may be finally adopted among these people. It is to be hoped that the Government will free their Hospitals Commission from the restriction that they have imposed upon it, contrary to the whole spirit of Lord Cave's Report.

